# Increased levels of YKL-40 in patients with diabetes mellitus: a systematic review and meta-analysis

**DOI:** 10.1186/s13098-021-00624-9

**Published:** 2021-01-15

**Authors:** Wanwan Luo, Lingmin Zhang, Lingling Sheng, Zhencheng Zhang, Zaixing Yang

**Affiliations:** Department of Laboratory Medicine, Huangyan Hospital of Wenzhou Medical University, Taizhou First People’s Hospital, Taizhou, Zhejiang China

**Keywords:** Diabetes mellitus, YKL-40, Diabetic nephropathy, Meta-analysis

## Abstract

**Background:**

Diabetes mellitus (DM) could be classified as type 1 diabetes mellitus (T1DM), type 2 diabetes mellitus (T2DM), gestational diabetes mellitus (GDM) and others according to etiology and pathology. Diabetic nephropathy (DN) is one of the most serious complications of DM. YKL-40 is a marker of inflammation and some studies have indicated that DM was related with inflammation. The objective of our study is to perform a systematic review and meta-analysis to confirm the relationship between YKL-40 and DM as well as DN.

**Methods:**

Pubmed, Embase, CNKI and Chinese wanfang databases were searched for eligible studies by two independent authors. Studies were included in this meta-analysis if they fulfilled the following inclusion criteria: (1) a study involving the role of YKL-40 in DM (or DN) designed as a case–control study or cohort study; (2) the data of serum YKL-40 levels were available; (3) studies were published in English or Chinese. Finally, twenty-five studies were included in this meta-analysis.

**Results:**

Compared with healthy controls, DM patients had significantly higher levels of YKL-40 (DM: SMD = 1.62, 95% CI 1.08 to 2.25, P = 0.000; GDM: SMD = 2.85, 95% CI 1.01 to 4.70, P = 0.002). Additionally, DM patients with different degree of albuminuria had significantly higher levels of YKL-40 compared with healthy controls (normoalbuminuria: SMD = 1.58, 95% CI 0.59 to 2.56, P = 0.002; microalbuminuria: SMD = 2.57, 95% CI 0.92 to 4.22, P = 0.002; macroalbuminuria: SMD = 2.69, 95% CI 1.40 to 3.98, P = 0.000) and serum YKL-40 levels increased with increasing severity of albuminuria among DM patients (microalbuminuria vs normoalbuminuria: SMD = 1.49, 95% CI 0.28 to 2.71, P = 0.016; macroalbuminuria vs microalbuminuria: SMD = 0.93, 95% CI 0.34 to 1.52, P = 0.002).

**Conclusions:**

Our current meta-analysis demonstrates that serum level of YKL-40 is increased in DM and positively associated with the severe degree of albuminuria. Therefore, we suggest that YKL-40 could be considered to be detected, along with other inflammatory markers, if DM, especially DN, is suspected.

## Background

Diabetes mellitus (DM) is a common disease in the modern society. According to etiology and pathology, DM could be classified as type 1 diabetes mellitus (T1DM), type 2 diabetes mellitus (T2DM), gestational diabetes mellitus (GDM) and others [1]. Diabetic nephropathy (DN), defined by low estimated glomerular filtration rate (< 60 mL/min/1.73 m^2^ for 3 months or more) or albuminuria (urinary albumin-to-creatinine ratio ≥ 30 mg/g) in the setting of DM [2], is one of the most serious complications of DM. Previous epidemiological studies have indicated that 25% to 40% of patients with T1DM and 5% to 40% of patients with T2DM ultimately develop DN [3, 4]. The pathology of DM is not totally understood. Some studies have indicated that DM is related to inflammation [5, 6]. Inflammatory markers, including interleukin (IL) 6, IL-1βand tumor necrosis factor (TNF)-α, were found increased in DM patients [7, 8].

YKL-40, also called human cartilage glycoprotein-39 (HCgp-39), is a 40 KDa heparin- and chitin-binding glycoprotein [9]. In vivo, CD 16 + monocytes are a source of YKL-40 and transcription factor Sp1 plays an important role in regulating of YKL-40 [10, 11]. In addition, YKL-40 is secreted by chondrocytes, synovial cells and neutrophils [9]. In vitro, YKL-40 is secreted by various cells, including vascular smooth muscle cells (VSMCs), activated macrophages and macrophages during late stages of differentiation [12]. We assume that there might be an association between DM and YKL-40 since YKL-40 is a new inflammatory marker. Recently, plenty of studies have explored the relationship of DM and YKL-40. But the conclusions of these studies were inconsistent, which might be associated with the sample sizes, methodology and so forth. The objective of our study is to perform a systematic review and meta-analysis to confirm the relationship between YKL-40 and DM as well as DN.

## Materials and methods

### Literature search

Pubmed, Embase, CNKI and Chinese wanfang databases were searched for eligible studies published before April 2020 using combinations of the following terms: diabetes; YKL-40; HC gp-39. All studies were retrieved by two independent reviewers and disagreements were solved by discussion.

### Study selection

Studies were included in this meta-analysis if they fulfilled the following inclusion criteria: (1) a study involving the role of YKL-40 in DM (or DN) designed as a case–control study or cohort study; (2) the data of serum YKL-40 levels were available (mean/standard deviation or median/range or median/interquartile interval was provided); (3) studies were published in English or Chinese. In case of duplicated data, only the most recent and complete study was included.

**Fig. 1:**
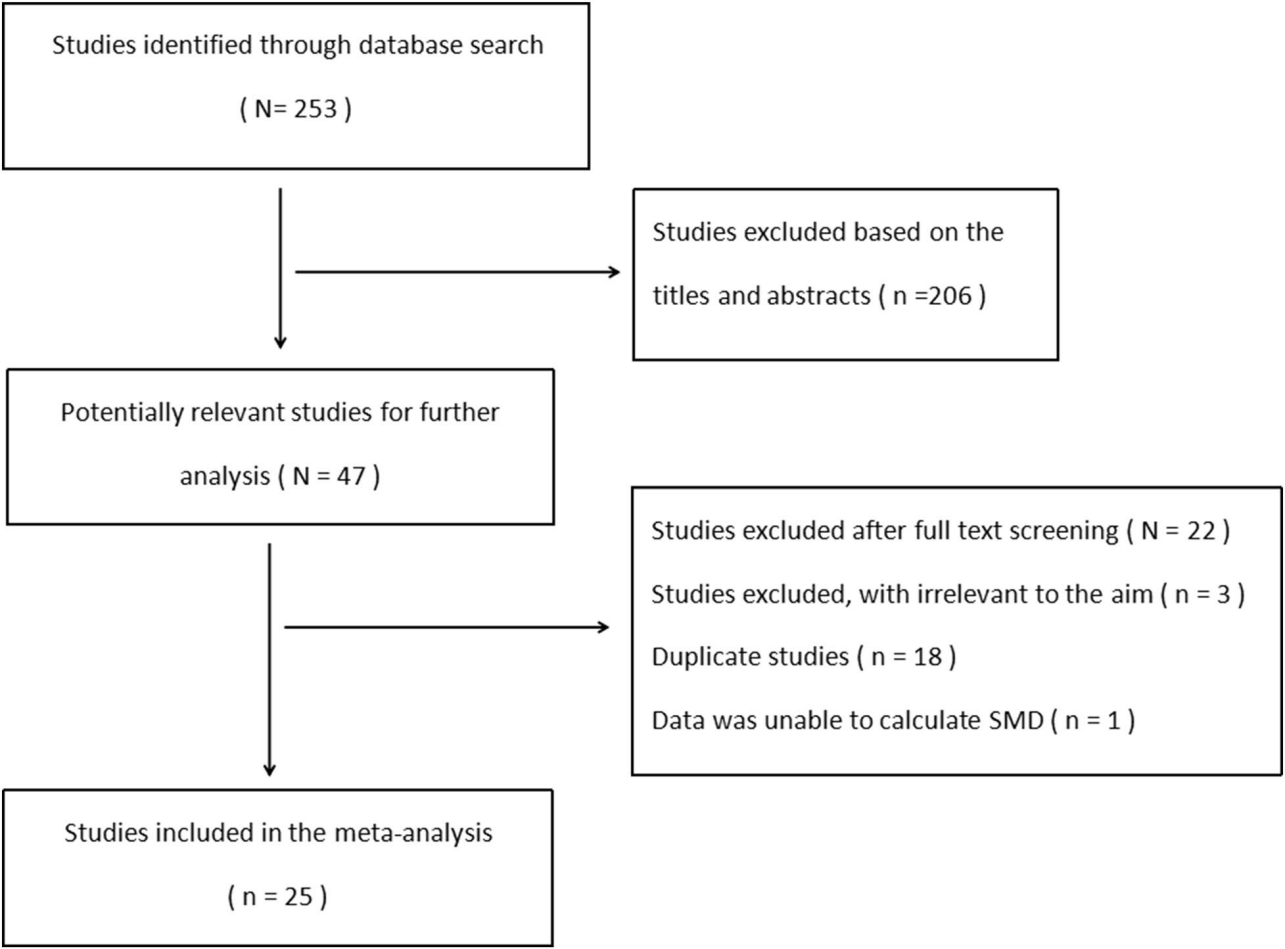
Flowchart of study selection

A total of 253 studies were identified in the initial search. Of these, 206 studies were excluded after screening on titles and abstracts. Full-text reading was performed only for 47 potential studies and details of the searches were shown in the flow chart (Fig. 1). Two publications [13, 14], written by the same authors, reported same population, so only the study [13] with more participants was included in our meta-analysis. Finally, 25 studies [13, 15–38] that met the inclusion criteria were included in this systematic review. And all the included studies are case–control studies. Of these, 14 studies were written in English and others were written in Chinese.

### Data extraction and statistical analysis

Some of the included studies provided YKL-40 concentration by median and range (or interquartile interval), which were converted to mean (SD) by estimation methods [39]. The statistical software R was used during the data estimation.

Standardized mean differences (SMD) with 95% confidence interval (CI) was calculated to compare the levels of serum YKL-40 in the DM (or DN) patients with the levels in healthy controls (P < 0.05 was considered statistically significant). The between-study heterogeneity was assessed by chi-square statistic and quantified by I-squared (I^2^). I^2^ values of 25%, 50% and 70% were considered as low, moderate and high heterogeneity, respectively [40]. The fixed-effects model was applied if I^2^ < 50%. Otherwise, the random-effects model was used. If a notable heterogeneity existed, the Galbraith plot was used to detect potential sources of heterogeneity [41]. Additionally, subgroup analyses were applied according to region, age and type of DM. To assess the stability of results, sensitivity analysis was performed by sequential omission of individual studies. Egger’s test and funnel plot were used to evaluate the presence of potential publication bias, and P < 0.05 was considered to represent statistically significant publication bias [42]. All statistical analyses were performed with STATA 12.0 software.

## Results

### Characteristic of included studies

Totally, 25 studies involving 2498 DM patients and 1424 healthy controls were included in our meta-analysis. Of these 25 studies, 12 were conducted for the different serum levels of YKL-40 between DM patients and healthy controls, 5 were analyzed for YKL-40 levels between GDM patients and healthy controls, and 8 were detected for YKL-40 levels between DM patients with different degree of albuminuria and healthy controls. The characteristics of the included publications are shown in Table 1.

**Table 1:** Characteristics of the studies included in this meta-analysis

Study	Region	Year	Type of DM	No. of DM patients	No. of healthy controls	Mean age of DM patients	Mean age of healthy controls	Diagnosis criteria	Method
Jian Li et al. [15]	China	2015	GDM	35	43	29.3 ± 3.1	30.6 ± 3.8	ADA 2012	ELISA (Quidel, USA)
Rathcke et al. [16]	Denmark	2005	T2DM	87	158	54.2 (40–70)^a^	NA	National Diabetes Data Group 1979	ELISA (Quidel, USA)
Thomsen et al. [17]	Denmark	2010	T2DM	45	20	54 (41–73)^b^	50 (34–66)^b^	NA	ELISA (Quidel, USA)
Schaller et al. [18]	Austria	2010	GDM	28	30	33 ± 6	33 ± 4	ADA criteria for GDM 2004	ELISA (Quidel, USA)
Sakamoto et al. [19]	Japan	2013	T1DM	131	97	24.7 ± 5.9	25.5 ± 2.7	NA	ELISA (Quidel, USA)
Rinnov et al. [20]	Denmark	2015	GDM	10	8	31.1 ± 5.6	28.1 ± 1.8	OGTT 2 h GLU ≥ 9.0 mM	ELISA (Quidel, USA)
Abd El Dayem et al. [21]	Egypt	2015	T1DM	62	30	16.32 ± 1.52	16.13 ± 2.63	NA	ELISA (Quidel, USA)
Shiasi et al. [22]	Iran	2017	T1DM	49	43	12.20 ± 3.86	10.95 ± 3.83	ADA	ELISA (Quidel, USA)
Rekha Kumari et al. [23]	India	2015	T2DM	30	30	44.4 ± 2.7	45.95 ± 3.4	NA	ELISA
Song Wei et al. [24]	China	2015	T2DM	210	210	58.29 ± 5.94	59.98 ± 7.53	NA	ELISA (Quidel, USA)
Ye Kejun et al. [25]	China	2016	GDM	50	50	27.2 ± 3.4	28.6 ± 3.8	ADA 2005	ELISA
Chen Qingfu et al. [26]	China	2014	T2DM	48	45	NA	48.1 ± 13.7	WHO 1999	ELISA (Quidel, USA)
Li Peng et al. [27]	China	2011	T2DM	41	40	54.61 ± 12.37	42.8 ± 13.52	NA	ELISA (Becton,Dickinson and Company, USA)
Lin Lijun et al. [28]	China	2019	T2DM	42	40	NA	NA	NA	ELISA
Xun Shengli et al. [29]	China	2017	GDM	60	20	27.85 ± 4.48	26.82 ± 3.10	Obstetrics and gynecology [M]	ELISA
Yu Yeye et al. [30]	China	2018	T2DM	60	60	46.48 ± 11.54	47.83 ± 9.68	ADA 2007	ELISA
Ren Lijue et al. [31]	China	2019	T2DM	30	30	57.20 ± 10.30	54.5 ± 10.44	WHO 1999	ELISA
Rathcke et al. [32]	Denmark	2009	T1DM	58^A^/46^B^/45^C^	55	55.6 ± 10.8^A^/54 ± 11.1^B^/49 ± 9.6^C^	50.5 ± 10.9	NA	ELISA (Quidel, USA)
Røndbjerg et al. [33]	Denmark	2011	T2DM	49^A^/35^B^/21^C^	20	61.3 ± 12.0^A^/60.1 ± 11.7^B^/64 ± 13.1^C^	57.1 ± 7.2	NA	ELISA (Quidel, USA)
Lee et al. [34]	South Korea	2012	T2DM	25^A^/25^B^/25^C^	22	55.6 ± 11.1^A^/57.0 ± 11.6^B^/56.0 ± 9.8^C^	52.4 ± 5.8	NA	ELISA
Han et al. [13]	China	2015	T2DM	260^A^/246^B^/232^C^	210	52.83 ± 4.30^A^/53.93 ± 4.56^B^/ 53.93 ± 4.22^C^	53.40 ± 4.28	ADA 2007	ELISA (Bio-Technology Co. Ltd., USA)
Umapathy et al. [35]	India	2018	T2DM	81^A^/73^B^/69^C^	83	54.07 ± 11.09^A^/55.1 ± 10.9^B^/57.39 ± 5.39^C^	52.59 ± 12.9	NA	a multiplex bead-based assay system (Bio-Rad, Hercules, California, USA)
Zhu Huijing et al. [36]	China	2015	T2DM	23^A^/24^B^/23^C^	20	63.00 ± 13.76^A^/65.33 ± 9.13^B^/66.35 ± 7.84^C^	62.0 ± 11.16	ADA 2007	ELISA
Wang Huan et al. [37]	China	2015	T2DM	21^B^/39^C^	30	NA	68 ± 8	NA	ELISA (Quidel, USA)
Yu Zhixuan et al. [38]	China	2017	T2DM	30^A^/30^B^	30	NA	55.45 ± 7.36	NA	ELISA

*T1DM* type 1 diabetes mellitus, *T2DM* type 2 diabetes mellitus, *GDM* gestational diabetes mellitus, *ADA*, American Diabetes Association, *WHO* World Health Organization, *ELISA* enzyme linked immunosorbent assay, *NA* Not available

^A^DM patients with normoalbuminuria,

^B^DM patients with microalbuminuria

^C^DM patients with macroalbuminuria

^a^Mean/range

^b^Median/range

### Data analysis

#### Association between serum YKL-40 levels and DM

**Fig. 2:**
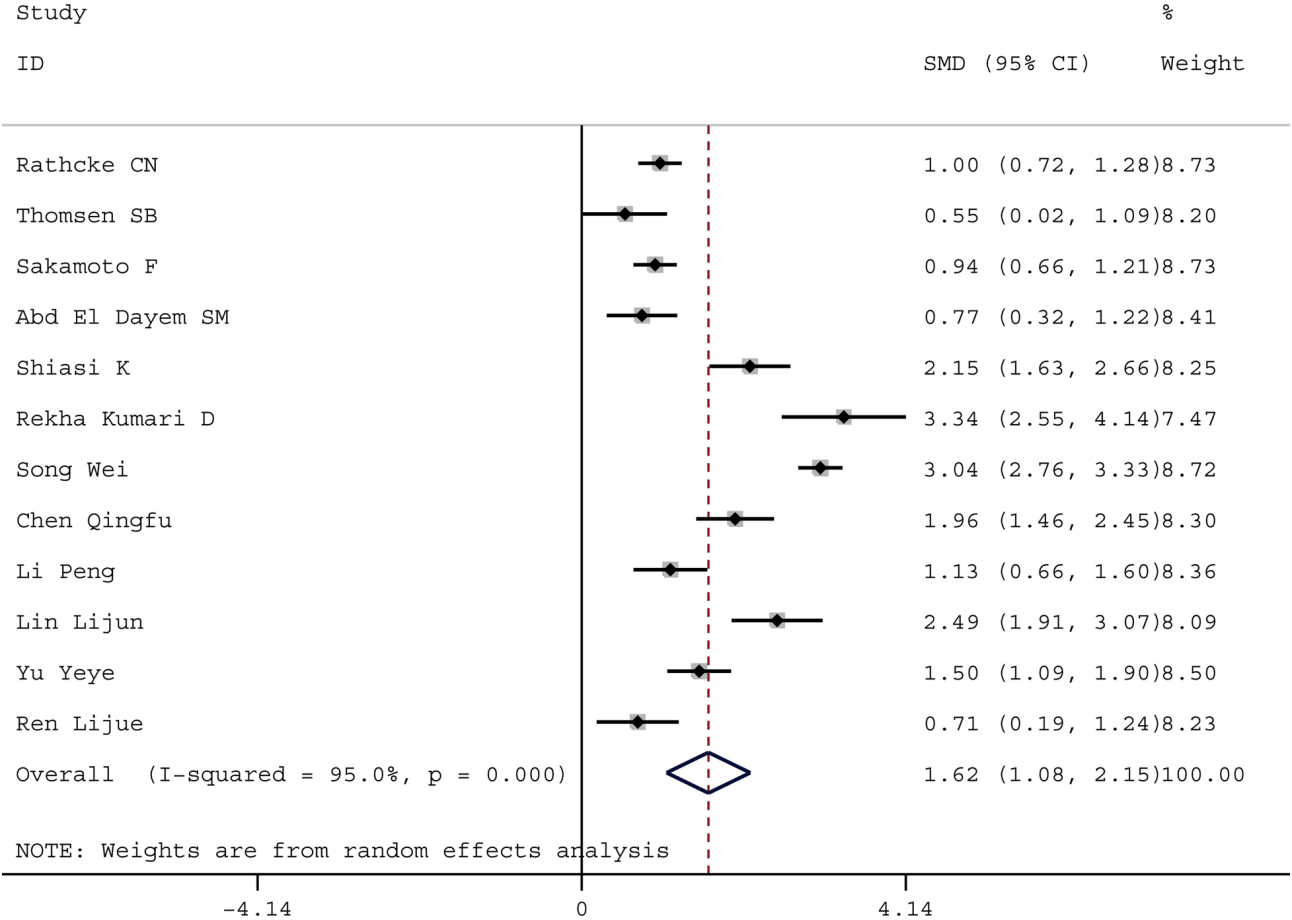
Forest plot of YKL-40 levels in DM patienst compared with healthy controls

**Fig. 3:**
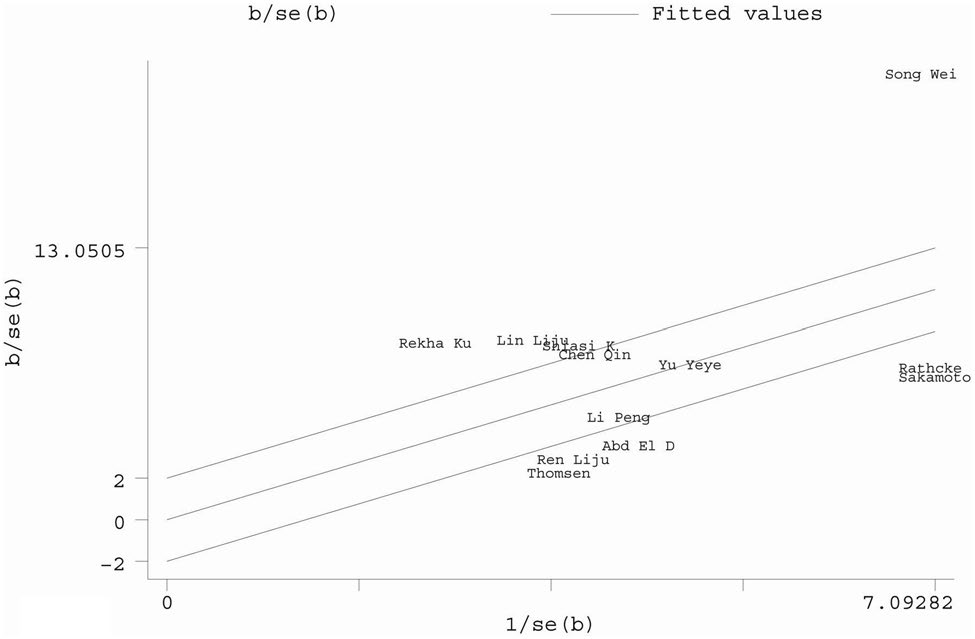
Galbraith plot of YKL-40 levels in DM patients compared with healthy controls

Totally, 12 studies showed an association between the serum YKL-40 levels and DM. The meta-analysis results indicated that the serum YKL-40 levels were significantly higher in DM patients compared with healthy controls (SMD = 1.62, 95% CI 1.08 to 2.25, P = 0.000) (Fig. 2). The Galbraith plot was used because of the notable heterogeneity. But the major source of heterogeneity could not be found since too many of the studies were outliers (Fig. 3). Furthermore, subgroup analyses by type of DM (supplementary material), region and age showed that YKL-40 levels were still higher in DM patients than those in healthy controls. The value of I^2^ remained high in various subgroups, with the exception of one subgroup for studies based on population of Asia.

#### Association between serum YKL-40 levels and GDM

**Fig. 4:**
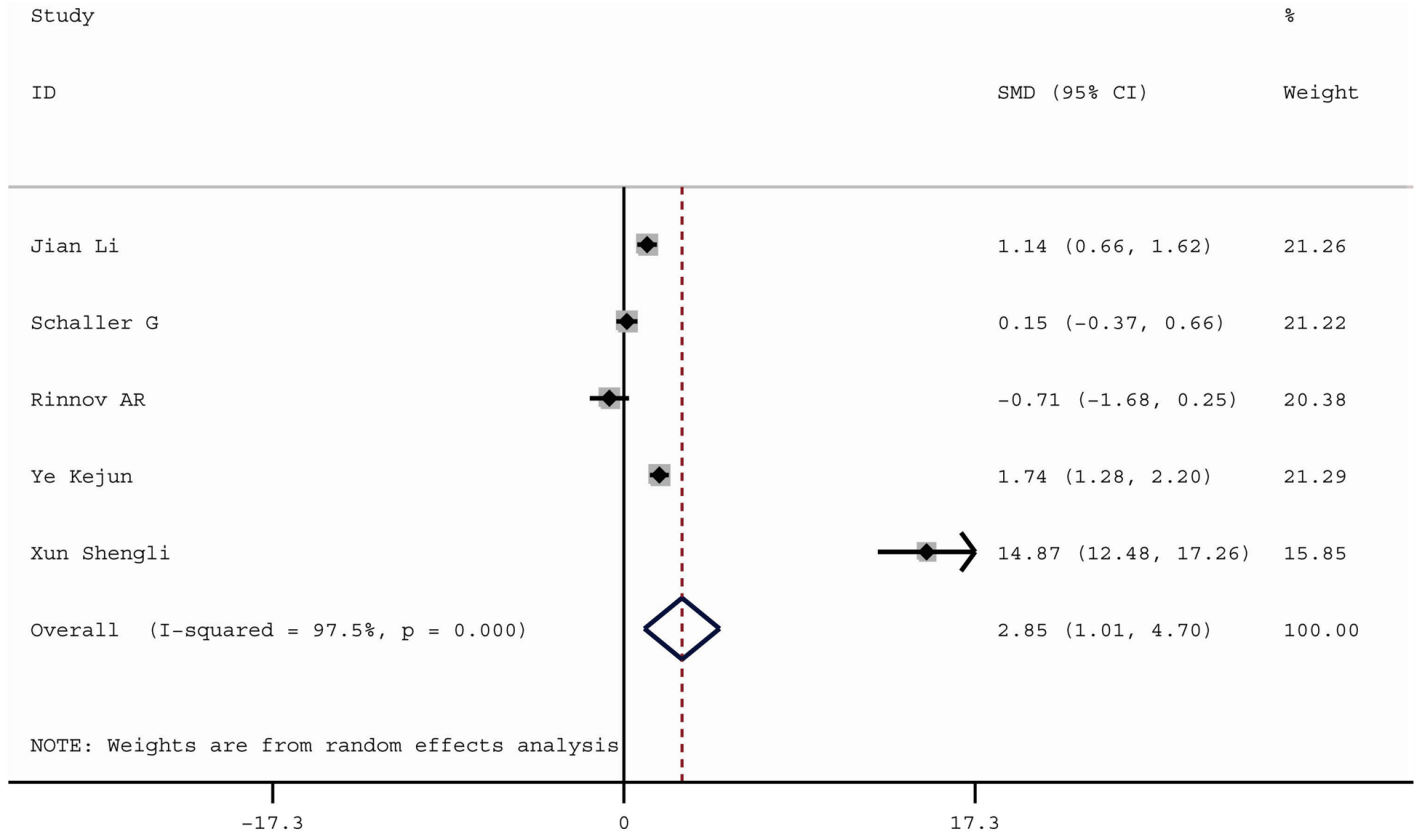
Forest plot of YKL-40 levels in GDM patients compared with healthy controls

Owing to significant heterogeneity, we used the random-effects model. The pooled SMD was 2.85 (95% CI 1.01 to 4.70, P = 0.002), which indicated that the serum YKL-40 concentrations were significantly higher in GDM patients compared with healthy pregnancies (Fig. 4). The source of heterogeneity was hard to be found by the Galbraith plot because the studies were too dispersive. However, when performing sensitivity analysis by sequential omission of individual studies, YKL-40 was not associated with GDM when the article by XunShengli et al. [29] was removed. The pooled SMD was 0.64 (95% CI − 0.28 to 1.56) (P > 0.05).

#### Association between serum YKL-40 levels and albuminuria in DM patients

There were 7, 8 and 7 studies analyzing the relationship between serum YKL-40 levels and normoalbuminuria, microalbuminuria and macroalbuminuria, respectively. The forest plot with a random-effects model showed that DM patients with different degree of albuminuria had significantly higher levels of YKL-40 compared with healthy controls (normoalbuminuria: SMD = 1.58, 95% CI 0.59 to 2.56, P = 0.002; microalbuminuria: SMD = 2.57, 95% CI 0.92 to 4.22, P = 0.002; macroalbuminuria: SMD = 2.69, 95% CI 1.40 to 3.98, P = 0.000). The Galbraith plot was applied to detect the potential source of heterogeneity. However, we could not find the possible source of heterogeneity because it plotted too many studies as the outliers. In addition, we conducted subgroup analyses by region and type of DM. The results did not change in various subgroups, and the value of I^2^ remained high in various subgroups, with the exception of one subgroup for studies based on population of Asia. What’s more, serum YKL-40 levels increased with increasing severity of albuminuria among DM patients (microalbuminuria vs normoalbuminuria: SMD = 1.49, 95% CI 0.28 to 2.71, P = 0.016; macroalbuminuria vs microalbuminuria: SMD = 0.93, 95% CI 0.34 to 1.52, P = 0.002).

#### Sensitivity analysis

We performed a sensitivity analysis by sequential omission of individual studies. When serum YKL-40 levels were compared between DM patients and heathy controls as well as DM patients with different degree of albuminuria and healthy controls, the pooled SMD were not materially altered. However, YKL-40 was not associated with GDM when the study by XunShengli et al.[29] was deleted.

#### Publication bias

Funnel plot and Egger’s test were conducted to evaluate the potential publication bias. There was no obvious funnel plot asymmetry and all the P values of the Egger’s tests were over 0.05, suggesting that publication bias was not evident in our meta-analysis.

## Discussion

To our knowledge, this is the first systematic review and meta-analysis to assess the relationship between YKL-40 and DM. Our study indicate that DM patients have a significantly higher level of YKL-40 compared with healthy controls. In addition, YKL-40 concentrations are higher in DM patients with different degree of albuminuria than those in healthy controls and increase with increasing severity of albuminuria.

Diabetes mellitus is a complex group of metabolic diseases characterized by hyperglycemia and is a major public health problem throughout the world. Both of T1DM and T2DM are genetic predisposition and influenced by environment. The genes responsible for T1DM are carried on chromosome 6p21 and take control of the immune system [43]. Many genes are relative to T2DM, but most of them have not been identified. Recently, inflammation is involved in the pathogenesis of DM. Previous study have found that long-term T1DM patients have a significantly higher level of CRP than healthy controls [44]. Besides, CRP is also higher in T2DM patients than in healthy controls [45]. But the role of inflammatory processes seems to be more important in the development of T2DM than T1DM. Some studies have indicated that inflammatory markers such as CRP and IL-6 are increased in healthy population who later developed T2DM [46, 47], suggesting that inflammation may occur ahead of the diagnosis of T2DM. Insulin resistance is common in T2DM and most patients with T2DM are obese, which itself can cause some degree of insulin resistance. Obesity, especially activation of adipose tissue, might enhance the release of inflammatory factors [48].

YKL-40, a new inflammatory marker, is related to both acute and chronic inflammation. Some studies have showed that levels of YKL-40 are increased in patients with purulent menigitis, rheumatoid arthritis, osteoarthritis, systemic lupus erythematosus and inflammatory bowel disease [49, 50]. Obesity is related to increased macrophage infiltration of adipose tissue and plays an important role in the development of insulin resistance [51]. YKL-40 is possibly with relation to the insulin resistance based on the macrophage infiltration and adipose tissue [12]. All the evidences above indicate that YKL-40 might have a relationship with DM. And our study, with more strong power, confirm that patients with DM have significantly higher levels of YKL-40 compared with healthy controls. What’s more, some studies have showed that inflammation is associated with hyperglycemia. And inflammatory markers, including IL-6, IL-1βand TNF-α, are often found increased in DM patients, whose glucose in poorly controlled [7, 8]. The great majority of studies (22 studies) included in our meta-analysis provided data of HhA1c values. In some studies, HbA1c values were higher than upper limits of normal range, but 5 studies [23–25, 27, 30] showed that YKL-40 levels are positively correlated with HbA1c, while 3 studies [19, 21, 22] did not show any correlation. Therefore, although YKL-40 is also an inflammatory cytokine, the relationship between hyperglycemia and YKL-40 needs to be confirmed.

The prevalence of GDM is increasing all over the world, of which the exact pathogenesis is not quietly understood. But many findings have showed that GDM patients have a trend of developing to T2MD. There are also some studies indicating that insulin resistance is an important pathophysiological contributor of GDM [52, 53]. Our present study find that the serum YKL-40 levels are higher in GDM patients than in healthy pregnancies. However, when the study by XunShengli et al. [29] is deleted during sensitivity analysis, YKL-40 is not associated with GDM. In the study by XunShengli et al., Enzyme Linked Immunosorbent Assay (ELISA) without details about the machine and reagents was used to measure the serum YKL-40 levels and the unit was pg/ml (ng/ml was used in other included studies). Compared with other included studies, the values of YKL-40 in this study were extremely small, which may have a strong contribution to obtain statistical significance. Anyway, the association between YKL-40 and GDM remains to be further confirmed by larger number of studies.

There are three types of complications of DM, including macrovascular, microvascular and neurologic. Kidney is the most obviously involved organ in microvascular complications and urinary albumin is a sign of DN. Some studies have found a high prevalence of microalbuminuria in DM patients [54, 55]. The pathogenesis of DN is multiple, and inflammation seems to be a major mechanism. Interaction of metabolism and hemodynamics, which activates many inflammatory molecules and pathway, results in DN [56, 57]. In addition, vascular endothelial dysfunction is a major factor in the pathogenesis of diabetic micro-angiopathy [58]. And YKL-40 is expressed in the development of endothelial dysfunction, during the differentiation and maturation of CD14 + monocytes to CD14−, CD16 + macrophages [12]. YKL-40, as a marker of inflammation and endothelial dysfunction, is found associated with albuminuria in T2DM patients [59, 60]. Consistent with previous studies, we find that the levels of YKL-40 are higher in DM patients with different degree of albuminuria compared with healthy controls and the levels of YKL-40 are positively related with the severe degree of albuminuria.

### Study limitations

Some limitations of this study should be mentioned. First, the heterogeneity is high and the major causes are not found by the Galbraith plot and subgroup analyses. Second, the criteria of normoalbuminuria, microalbuminuria and macroalbuminuria were different among the studies included in this meta-analysis. In some studies, urinary albumin excretion rate was used as classification criterion, but in others, albumin/creatinine was used. As thus, the results of our study are not stable enough.

## Conclusion

In summary, our current meta-analysis demonstrates that serum level of YKL-40 is increased in DM and positively associated with the severe degree of albuminuria. Therefore, we suggest that YKL-40 could be considered to be detected, along with other inflammatory markers, if DM, especially DN, is suspected.

## Data Availability

The datasets used and/or analysed during the current study are available from the corresponding author on reasonable request.
